# Development and Transferability of EST-SSR Markers for *Pinus koraiensis* from Cold-Stressed Transcriptome through Illumina Sequencing

**DOI:** 10.3390/genes11050500

**Published:** 2020-05-02

**Authors:** Xiang Li, Xiaoting Liu, Jiatong Wei, Yan Li, Mulualem Tigabu, Xiyang Zhao

**Affiliations:** 1 State Key Laboratory of Tree Genetics and Breeding, School of Forestry, Northeast Forestry University, Harbin 150040, China; lx2016bjfu@163.com (X.L.); 15754638841@163.com (X.L.); wwwjttt@163.com (J.W.); Ly2019nefu@163.com (Y.L.); 2 Southern Swedish Forest Research Center, Swedish University of Agricultural Sciences, SE-230 53 Alnarp, Sweden; Mulualem.Tigabu@slu.se

**Keywords:** *Pinus koraiensis*, microsatellite markers, population structure, RNA-Seq, interspecific transferability

## Abstract

*Pinus koraiensis* has significant economic and ecological value in Northeast China. However, due to the lack of suitable molecular markers, only a few available microsatellite markers were developed for further population genetics studies. In this study, for the first time we developed expressed sequence tag–simple sequence repeat (EST-SSR) markers from the cold-stressed transcriptome of *P. koraiensis* using Illumina Sequencing. We identified a total of 7,235 EST-SSRs from 97,376 sequences, and we tested their transferability among seven related Pinus species. The results showed that trinucleotides were the most abundant type of repeat (1287, 18.74%) excluding mononucleotides, followed by dinucleotides (1284, 18.7%) and tetranucleotides (72, 1.05%). The most dominant dinucleotides and trinucleotide repeat motifs were AT/AT (535, 7.79%) and AAT/ATT (103, 1.5%). The observed heterozygosity (Ho) and expected heterozygosity (He) ranged from 0.002 to 0.986 and 0.017 to 0.743, respectively, and the polymorphism information content (PIC) values and number of alleles (Na) varied from 0.029 to 0.794 and 2 to 23, respectively. A total of 8 natural *P. koraiensis* populations were divided into two main genetic clusters. Furthermore, nine of twenty polymorphic primer pairs were successfully amplified in seven Pinus species, and at least 80% of the successful *P. koraiensis* EST-SSR primers could be amplified in more than four species (16, 80%). Combined results for the development of EST-SSR markers in *P. koraiensis* and transferability among related species would contribute to improved studies on the genetic diversity and population structure in *P. koraiensis* and phylogenetic relationships among Pinus species. They would also provide a significant source for quantitative trait locus analysis.

## 1. Introduction

*Pinus koraiensis*, an evergreen conifer species, is an important native and precious forest tree in Northeastern China, as well as in the far eastern region of Russia and the Korean peninsula [1,2]. *P. koraiensis* has great ecological and economic value, and thus plays a significant role in ecological environmental construction and economic development in China. For centuries, *P. koraiensis* has been extensively used as a major source of excellent timber, natural remedies and edible pine nuts, due to its excellent wood properties and nutritional value [3,4,5]. Pine nuts have a high nutritional value, and they include a variety of essential nutrients, such as vitamins, proteins, and unsaturated fatty acids, among others [6,7]. The nuts have the function of preventing cardiovascular disease caused by elevated cholesterol [8]. Moreover, *P. koraiensis* is also considered a main ecological and pioneer tree species in China, particularly in the northeast forest areas, and it has a significant effect on eco-environmental construction [9]. Remarkably, the Xiaoxinganling Mountain region in Yichun, in Northeastern China has excellent natural conditions, wherein more than half of the natural *P. koraiensis* in the world is intensively distributed. Currently, because of the great increase in demand for timber resources, after more than 50 years of logging, the number of natural *P. koraiensis* germplasm resources has decreased sharply, and in 2013, *P. koraiensis* was listed as a vulnerable species by the International Union for Conservation of Nature (IUCN) [10]. Therefore, to protect and utilize precious wild resources of *P. koraiensis*, it is essential to evaluate the genetic diversity and genetic structure of natural germplasm resources, which provide useful genetic information for the protection of elite wild resources and for further research on the origin and evolution of this coniferous species and its related species. However, due to a lack of genome information, only a limited number of polymorphic molecular markers, such as simple sequence repeats (SSRs), have been developed and used in population genetics studies, which has greatly hindered the application of molecular marker technology in the study of genetic improvement of *P. koraiensis* [11,12].

The use of molecular markers, especially microsatellite marker (SSRs), has become a significant genetic method for population genetics, genetic map construction and trait association studies, in many types of animal and plant breeding research [13]. Additionally, they are widely distributed in the genomes of various eukaryotes. With the rapid advancement of high-throughput sequencing technology, abundant expressed RNA sequence data from different tissues in many species have been sequenced and widely used in functional analyses of the development of important genes and molecular markers [14]. Identifying and developing a large number of molecular marker loci and their highly polymorphic primers using whole-genome and transcriptome data, are crucial for the protection and utilization of germplasm resources. Compared to other DNA molecular markers, such as the amplified fragment length polymorphism (AFLP), single nucleotide polymorphism (SNP), random amplified polymorphic DNA (RAPD), and restriction fragment length polymorphism (RFLP), SSRs are useful and efficient molecular markers with a high level of polymorphic information, low cost, and codominant characteristics; thus, they are considered a powerful tool to study genetic diversity, determine genetic structure, construct genetic maps, and identify relationships between many non-model species, especially endangered species [15,16,17]. Previous studies of SSR development, including chloroplast microsatellites (cpSSRs) and expressed sequence tag-simple sequence repeats (EST-SSRs) of *P. koraiensis,* were mainly based on multi-omics data such as the chloroplast genome and transcriptome [12,18,19]. However, because of the limitation of the whole *P. koraiensis* genome with a high heterozygosity level and sequence size, only a few available molecular markers have been developed and used in population genetic structure and genetic diversity studies [20,21,22]. Furthermore, the transferability and application of EST-SSR markers underlying different Pinus plants currently raise a few difficulties that remain to be addressed. Particularly, due to the lack of effective microsatellite markers in many Pinus species, relatively few studies have examined the genetic diversity and genetic relationship of plant germplasm resources. Considering that these markers demonstrate limited genetic information, it is urgent to develop a large number of high-quality molecular markers based on large amounts of sequencing data, which would provide a strong theoretical basis for tree genetic breeding, species identification, and gene localization in many model and non-model plants.

In this study, we used a set of cold-stressed transcriptome data through Illumina sequencing from *P. koraiensis*, a precious fruit-timber tree species in Northeastern China, and we successfully selected 20 ideal EST-SSR markers that were highly polymorphic and applicable to study the genetic diversity and population structure of natural *P. koraiensis*. Simultaneously, we also analyzed the amplification efficiency and transferability of EST-SSR markers to related species of *P. koraiensis.* The present research would help to deepen our understanding of genetic diversity and genetic relationship of *P. koraiensis* and accelerate the molecular breeding progress of this tree species.

## 2. Materials and Methods

### 2.1. Transcriptome Data Capture

The cold-stressed transcriptome data used in this study originated from the study in our laboratory by Wang et al. [23], and the relative transcriptome data was uploaded to the Sequence Read Archive (SRA) public database (No. PRJNA510863) at the National Center for Biotechnology Information (NCBI).

### 2.2. Plant Materials and DNA Extraction

**Figure 1:**
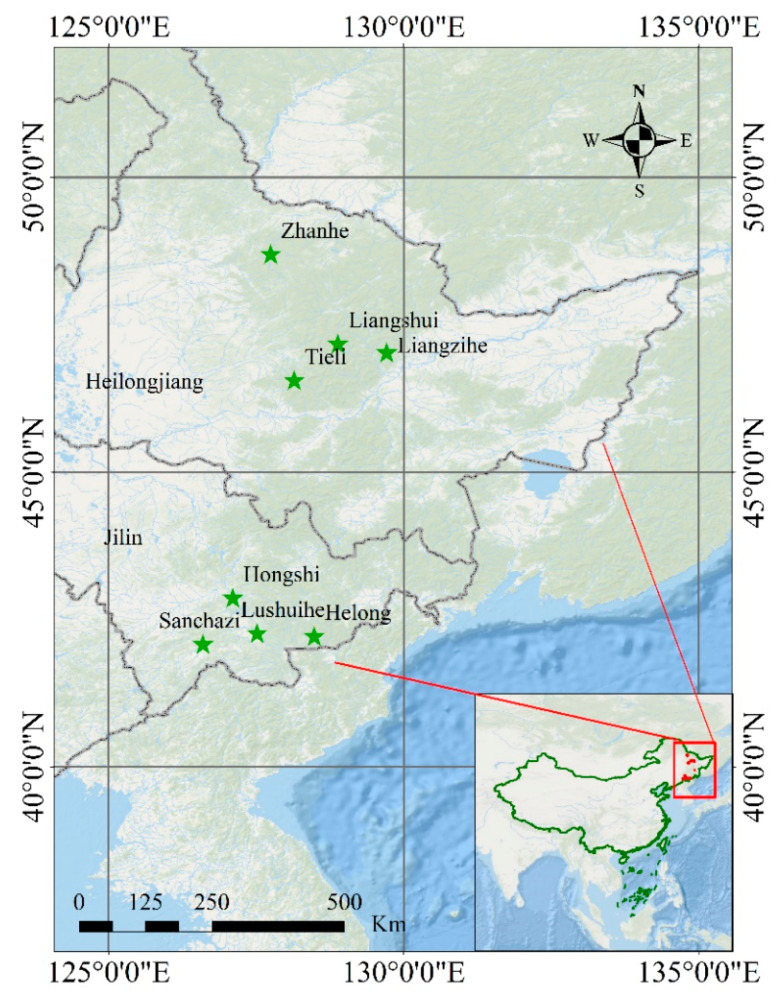
Distribution of the collected samples.

From 2018–2019, plant samples used to assess the availability and development of EST-SSR were collected from 8 natural populations of *P. koraiensis* from the cities of Tonghua (41°40′12″ N, 125°56′24″ E) and Helong (42°32′24″ N, 129°0′36″ E) in the Jilin province; and from Yichun (47°1′47″ N, 129°1′12″ E); Jiamusi (46°43′48″ N, 129°54′36″ E); Heihe (49°14′24″ N, 127°31′48″ E) in the Heilongjiang province (Figure 1). To verify and screen the polymorphic primers, ten representative adult trees in each populations were randomly selected, and all sampled individuals from eight populations were spaced at least 100 m apart. In total, 80 individuals from eight natural *P. koraiensis* population were collected in this study, including Zhanhe (ZH), Liangshui (LS), Tieli (TL), Liangzihe (LZH), Hongshi (HS), Sanchazi (SCZ), Lushuihe (LSH) and Helong (HL) (Table S1). The sampled individuals were distributed in the main natural geographical distribution region of *P. koraiensis*. The collected samples were immediately frozen in liquid nitrogen and then stored at −80 °C. Total DNA was extracted from the fresh needles of 80 individuals, according to the hexadecyl trimethyl ammonium bromide (CTAB) method. The DNA integrity and concentration were further determined by 1% agarose gel electrophoresis and using a K5500Plus micro-spectrophotometer (KAIAO technology development CO. Ltd., Beijing, China), respectively. Finally, the total DNA was diluted to the desired working concentration (25 ng/μL) and stored at −20 °C for PCR analysis, to validate the EST-SSR reliability.

### 2.3. EST-SSR Locus Detection and Primer Design

The detection and localization of potential SSRs were searched and implemented from 97,376 unigenes (75,061,632 bp) using the MISA tool (http://pgrc.ipk-gatersleben.de/misa/misa.html) [24]. The identification standards of minimum repeats of the SSR motif contained ten mononucleotide, six di-nucleotide, and five tri-, tetra-, penta-, and hexanucleotide motif repeats. Particularly, SSR motifs containing one mononucleotide were considered to be associated with a high risk of error during synthesis of the PCR product and were ultimately removed from the study. All primers for the SSR locus were designed using Primer3 software (http://primer3.sourceforge.net/releases.php). The main stated selection criteria for screening the primers were as follows—GC content from 45–55% with 55% as the optimum; primer length from 18–24 bp with 23 bp as the optimum length; annealing temperature between 55 and 65 °C, with 60 °C as the optimum temperature; and PCR product size ranging from 100 to 300 bp.

### 2.4. PCR Amplification and SSR Validation

To validate the quality of primer pairs, a total of 300 primer pair sequences were randomly selected and synthesized by Beijing Genomics Institute Tech Solutions (Bejing Liuhe) Co., Ltd. (Beijing, China) and used for PCR amplification in a 20-μL volume that included 10 μL 2 × Super PCR Mix (with green dye) (Beijing Genomics Institute Tech Solutions (Bejing Liuhe) Co., Ltd., Beijing, China), 4 μL M13 universal primer (1 μM, 5-TGTAAAACGACGGCCAGT-3), 5′ end addition of four fluorescent dyes to all forward primers (ROX, HEX, FAM and TAMRA), 2 μL genomic DNA (25 ng/μL), 0.8 μL forward primer (1 μM), and 3.2 μL reverse primer (1 μM). The PCR amplification conditions were as follows—94 °C for 5 min, followed by 30 cycles at 94 °C for 30 s, 57 °C for 30 s, and 72 °C for 30 s, followed by 8 cycles at 94 °C for 30 s, 55 °C for 30 s, and 72 °C for 30 s, and then a final extension at 72 °C for 10 min, using a TC-96/G/H(b)B Thermal Cycler (Hangzhou Bioer Technology Co., Ltd., Hangzhou, China). Finally, all primer pairs were used for amplification from eighty samples representing different populations for the development and assessment of EST-SSR polymorphisms.

### 2.5. Statistical Analyses

GeneMapper (version 4.1) was used to analyze the original data obtained using high-performance capillary electrophoresis (HPCE). GeneAlEx (version 6.5) [25] was used to calculate the population genetic parameters of each primer pair, including the number of alleles per locus (Na), observed heterozygosity (Ho), expected heterozygosity (He), and effective number of alleles (Ne). Information index and polymorphism information content (PIC) values were calculated using the Cervus software (version 3.0) [26]. In addition, the principal component analysis (PCA) was also performed in GeneALEX (version 6.5). To determine the genetic variation of populations including within populations, among populations within groups and among groups, the analyses of molecular variance (AMOVA) function in GeneAlEx version 6.5 was applied. In total, 999 random permutations were implemented for each sample in the GenAlEx software. The population genetic structure of eight *P. koraiensis* natural populations was analyzed by the STRUCTURE software (version 2.3.4) [27], based on Bayesian analysis. Ten independent runs were performed for each K value. In brief, the best K value was determined by the method described by [28]. The burn-in period iterations and Markov chain Monte Carlo repetitions for each run (K ranged from 1 to 8) were 100,000 each. The results obtained from STRUCTURE were further analyzed with the software of STRUCTURE HARVEST [29].

### 2.6. EST-SSR Primer Transferability in Related Species

EST-SSR primer pairs developed from the EST sequences had higher transferability in related species than SSRs in genomic DNA. In the present study, a total of seven *Pinus* species were used to detect and verify the transferability of 20 EST-SSR primer pairs in related species, including two-, three-, and five-needle pines. The species used in this study were as follows—*Pinus tabuliformis* and *Pinus sylvestris* collected from Northeast Forestry University Campus, Harbin, China (45°43′11″ N, 126°38′24″ E); *Pinus wallichiana* and *Pinus bungeana* sampled from the Chinese Academy of Forestry Sciences arboretum (40°0′36″ N, 116°14′23″ E); *Pinus pumila* and *Pinus sibirica* (51°38′32″ N, 127°27′12″ E) selected from Xinganling Mountains, Hulunbeier, China; *Pinus parviflora* sampled from Xuanwu Lake, Nanjing, China (32°5′53″ N, 118°48′36″ E); *Pinus armandi*, *Pinus yunnanensis*, and *Pinus khasys* collected from the Southwest forestry university arboretum, Kunming, China (25°4′12″ N, 102°46′12″ E). In this study, 32 samples were used to test the transferability of EST-SSR primers in Pinus species, and the samples collected from *Pinus sibirica*, *Pinus pumila*, *Pinus wallichiana*, *Pinus parviflora*, *Pinus bungeana*, *Pinus tabuliformis*, and *Pinus sylvestris* was 5, 5, 5, 2, 5, 5, and 5, respectively. The DNA extraction and PCR amplification methods for all *Pinus* species were performed as described above, with appropriate adjustments of the annealing temperature, template DNA concentration, and cycle times.

## 3. Results and Discussion

In total, 97,376 sequences containing 7235 SSRs were detected and identified from the transcriptome data, with 509 EST sequences containing more than one SSR and 354 (4.9%) SSRs present in the compound formation. Furthermore, the EST-SSR frequency was 6.84%, and the distribution density was 75.06 per Mb. The tri-nucleotide (1287, 18.74%) was the most abundant type of repeat excluding the mononucleotide, followed by the di-nucleotide (1284, 18.7%), tetra-nucleotide (72, 1.05%), hexa-nucleotide (53, 0.77%), and penta-nucleotide (25, 0.36%) (Table 1). In total, the repeat types were mainly distributed from mono- to tri-nucleotides, which accounted for 97.82% of the SSRs. This result was similar to the findings reported by Zhang et al. [30]. The frequencies of EST-SSRs for different tandem repeats are shown in Table 2. The lowest repeat motif for each SSR was five, and the largest number of tandem repeats was ten (1864, 27.14%), followed by 11 (937, 13.64%), 5 (929, 13.53%), 6 (709, 10.32%), 12 (530, 7.72%), 7 (346, 5.04%), and 13 (345, 5.02%); the other tandem repeats were less than 20%. The main tandem repeats were 10–13, reaching 53.52% of the SSRs (Table 2). The main motif type was 40 in 7235 SSRs. Generally, the EST-SSRs in many plants are mainly dinucleotides and trinucleotides, with the exception of single nucleotides. Remarkably, A/T (4090, 59.55%) was the dominant motif, followed by AT/AT (535, 7.79%) and TA/TA (394, 5.74%), which accounted for 73.08% of the total number of SSRs (Table 3). These results were similar to those found by Jia, which further support the conclusion that A/T and AT/AT are the main unit types (accounting for >50%) in *P. koraiensis* [31]. Among the SSRs, 535 and 103 contained the most abundant di- and tri-motif types, respectively. In contrast, the numbers of CG/CG and ACT/AGT were very few with approximately equal frequencies, which might indicate that CG/CG and ACT/AGT are rare in gymnosperm plants [32].

To determine the amplification efficiency of EST-SSR, we previously found that because of the influence of primer dimers, annealing temperature and number of introns, some EST-SSRs usually failed to amplify the expected product. In the present study, three hundred 1,884 primer pairs were randomly selected to detect and evaluate their polymorphisms across 4 individuals from different geographical populations in five regions. Furthermore, 96 (32%) of 300 amplified single and bright DNA bands (Figure S1) were further used to detect the polymorphism level across 16 *P. koraiensis* individuals sampled from five main distribution regions in Northeast China and 119 (39.67%) amplified PCR products that were larger or smaller than expected. The amplified PCR products were not found in 85 (28.33%) primer markers at various annealing temperatures. Using the same DNA templates, the results showed that 20 (6.7%) of the 96 amplified single and bright DNA bands successfully amplified the expected product size and could be used to conduct the population and conservation genetics studies; the remaining primer pairs were identified as monomorphic. Of the 20 amplified EST-SSRs, the di- (10, 50%) and tri-nucleotides (10, 50%) had the same number of repeat type and polymorphism frequencies; the other types of repeat units were not found in the present study.

The polymorphism parameters and sequence information for the amplified ESR-SSRs among 80 natural *P. koraiensis* individuals are listed in Table 4. The fragment sizes of the amplified products ranged from 188 bp to 275 bp. In total, 129 alleles in eighty individuals were detected, and the number of alleles generated using the 20 pleomorphic EST-SSRs primer pairs among the eight collected populations was between 2 (NEPK-34, NEPK-53 and NEPK-150) and 23 (NEPK-168), with an average of 6.45. The Ne was 0.894 (NEPK-34)–2.609 (NEPK-117), averaging 1.812 across all loci. The value of I was 0.032 (NEPK-34)–2.063 (NEPK-168), with an average of 0.579. The Ho ranged from 0.02 at locus NEPK-53 to 0.986 at locus NEPK-117, averaging 0.299 across all loci. Generally, one species with a high homozygosity rate can easily lead to the degradation and extinction of this species, which is not conducive to species reproduction. The He ranged from 0.017 (NEPK-34) to 0.743 (NEPK-168), with an average of 0.311 across all loci, showing a moderate homozygosity rate. The polymorphism information content (PIC) is mainly determined by the number of alleles and their distribution frequency, and it can be used to measure the degree of polymorphism of different loci and to further evaluate the molecular marker polymorphism detection ability. Among the parameters, the PIC value ranged from 0.029 (NEPK-150) to 0.794 (NEPK-145) across all loci, with eight of 20 loci showing a high level of informativeness (PIC > 0.50) and seven showing moderate informativeness (0.50 > PIC > 0.25). The average PIC value was 0.404. Locus NEPK-145 and NEPK-34 had the highest and lowest genetic diversity, respectively, with values of 0.513 and 0.017 for He and 0.794 and 0.032 for PIC, respectively. These results showed a high degree of genetic variation of each locus within the population, which is of great practical significance for the evaluation and selection of *P. koraiensis* germplasm resources. In general, the genetic diversity level of a species is affected by a number of factors, including the biological characteristics, population size, number of genetic markers, and geographical distribution range [33,34,35]. The twenty polymorphic EST-SSR primer pairs were developed based on the cold-stressed transcriptome of *P. koraiensis*, and a relatively moderate level of genetic diversity was detected using twenty EST-SSR markers in eighty *P. koraiensis* natural individuals, with mean Na, He, and PIC values of 6.45, 0.311, and 0.404, respectively. These results were similar to those found in fifty-three *P. koraiensis* germplasm resources of four seed orchards (I = 0.654, PIC = 0.325), as determined by six pairs of highly polymorphic EST-SSR primers [36]. However, in this study, the genetic diversity of *P. koraiensis* was lower than sixty open-pollinated families (I = 0.868, PIC = 0.450), determined using sixteen pairs of EST-SSR markers [31], and 204 samples of seven natural populations determined by nine polymorphic primers (Na = 6.7, He = 0.610) [12]. The main reason for this finding was that only 10 individuals for each natural populations were used to detect the genetic diversity level. Another reason might be differences in population types. Generally, the genetic diversity within cultured populations might maintain a relatively lower level compared with natural populations. The genetic diversity of *P. koraiensis* in this study was lower than naturally distributed Pinus species, including *Pinus tabulaeformis* [37] (mean Na = 6.52, He = 0.68 for 747 individuals from 29 populations), *Pinus yunnanensis* [38] (mean Na = 4.10, He = 0.43 for 20 populations), *Pinus dabieshanensis* [30] (mean Na = 3.70, He = 0.36 for 64 samples from 4 populations) and *Pinus bungeana* [39] (mean Na = 3.70, He = 0.36 for 476 samples from 5 distribution regions). Here, the reasons for the differences in levels of genetic diversity among different species included the following—(1) the mating mode, e.g., pollination methods used for the breeding population would affect the level of genetic diversity, where generally, with the increase in the self-crossing rate, the population genetic diversity tended to decrease; (2) genetic drift caused a loss or fixation of alleles, which would lead to a decline in the population genetic diversity; and (3) artificial and natural selection. In addition, the genetic diversity of species in narrow natural distribution areas, such as local varieties, endangered species, and endemic species, was generally lower than species with wide natural distribution areas. In our study, *P. koraiensis* was only naturally distributed in Northeast China, but it had a relatively moderate level of genetic diversity. The main reason for this phenomenon is that more than half of *P. koraiensis* worldwide is naturally distributed in Northeast China, with abundant genetic resources.

Recently, EST-SSR markers developed using expressed sequence tags have been widely used in the fields of genetic diversity, phylogenetic development, and molecular marker-assisted breeding. At present, the development of universal EST-SSR primers is a cheap and simple approach for species without whole genome and developed microsatellite loci [40]. However, for some species with complex genetic background, such as non-model species, genome-free species, and endangered species, traditional methods for SSR marker development are complex and expensive. Previous studies have found that because of the conservative lateral sequence of microsatellite DNA, EST-SSR primers have good transferability among close and even distantly related species [41,42]. With these crucial characteristics, they have been used for comparative genomics and phylogenetic analysis in several species, such as *Theobroma cacao* [43], *Chrysanthemum morifolium* [44], *Elymus sibiricus* [45] and *Taxus mairei* [46]. In the present study, 20 successful EST-SSR primers were further tested in Pinus relatives to test the transferability of EST-SSR primers (Table 5). Different genetic relationship showed different levels of transferability for *P. koraiensis* EST-SSRs. The majority of *P. koraiensis* EST-SSR primers generated a relatively high amplification efficiency ranging from 0% to 100% in Pinus species. There were 9 (NEPK-40, NEPK-32, NEPK-53, NEPK-72, NEPK-43, NEPK-38, NEPK-150, NEPK-179 and NEPK-184) and 1 (NEPK-34) primer pairs that generated the highest and lowest amplification efficiency, respectively; the other 10 primer pairs showed a moderate level of amplification efficiency. At least 80% of the successful *P. koraiensis* EST-SSR primers could be amplified in more than four of the Pinus species (16, 80%). Interestingly, at least 70% (5, 71%) of the tested Pinus species had a relatively high level of EST-SSR transferability (more than 70%). Compared to three- and two-needle pine, three five-needle pines showed dramatical levels of transferability (greater than 80%). The main reasons for these cases were as follows—(1) the transferability of EST-SSR primers is related to the genetic relationship between two species; (2) *Pinus sibirica*, *Pinus pumila*, and *Pinus wallichiana* belong to the five-needle group and are widely assumed to have a closer genetic relationship than two- and three-needle species. These results revealed high genome homology among seven Pinus species, and nine of 20 newly developed EST-SSRs from cold-stressed transcriptome of *P. koraiensis* could be used as universal markers in the Pinus species for further population genetics studies in related species and the identification of functional genes with significant economic traits.

**Figure 2:**
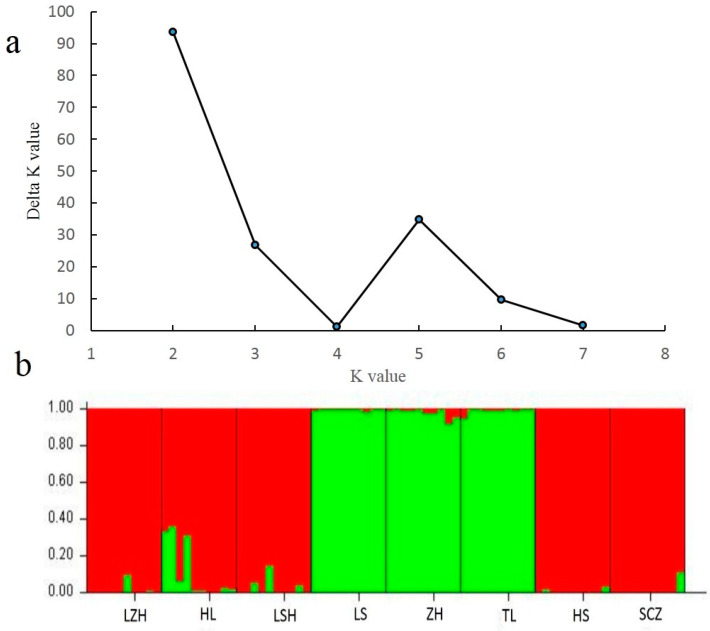
Results of STRUCTURE analysis for 80 individuals using 20 EST-SSR markers. (**a**) Estimation of population using Delta K value with cluster K ranging from 1 to 8. (**b**) Estimation of population structure based on STRUCTURE analysis.

**Figure 3:**
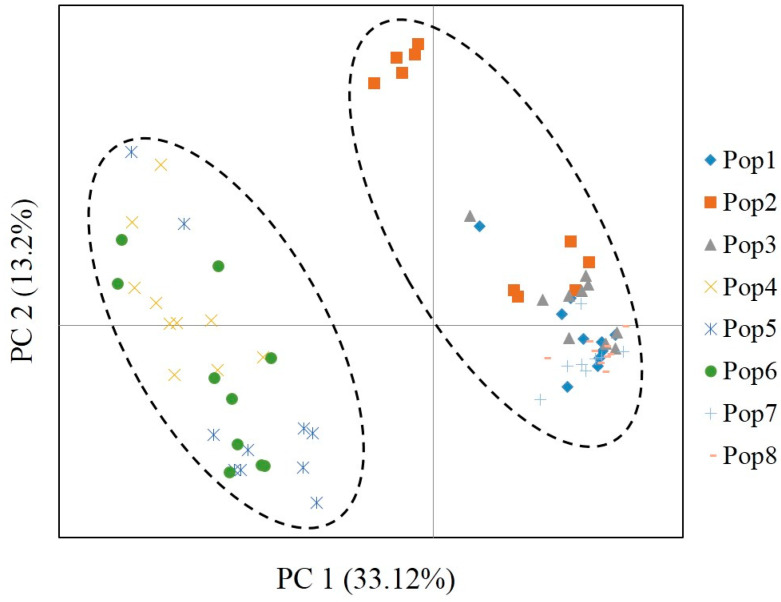
Principal component analysis based on 20 EST-SSR markers in *P. koraiensis*.

The population genetic structure of 80 individuals from 8 *P. koraiensis* natural populations was analyzed by the STRUCTURE 2.3.4 software. In this study, the number of cluster was set from 1 to 8 with 10 repetitions for each run. According to the STRUCTURE assignments, the optimal K value was observed at K = 2 with maximum Delta k value (Figure 2a); all collected individuals were further divided into two clusters (C1 and C2) (Figure 2b). C1 contained three populations (LS, ZH, and TL), and C2 contained five populations (LZH, LSH, HL, HS, and SCZ). Furthermore, the PCA analysis based on 20 EST-SSR markers was used to further evaluate the population genetic structure and demonstrated that the sampled *P. koraiensis* individuals were well-grouped into two groups based on the first two principal coordinates (Figure 3). In which, coordinates 1 explained 33.12% of the total variation, and coordinate 2 explained 13.2% of the total variation. The PCA results were consistent with the results of the structure analysis using the same EST-SSR markers and individuals. Notably, previous study reported that the seven natural *P. koraiensis* populations in Northeast China were also divided into two main groups, including two northern populations and five southern populations [12]. However, in our study, although the LZH *P. koraiensis* population were closer to the Xiaoxinganling Mountains populations (the northern population), it was eventually divided into the Changbaishan Mountains populations (the southern populations), based on the STRUCTURE and PCA analysis. The main reasons for this were: (1) The LZH population was located on the border between the Xiaoxinganling Mountain and Sanjiang Plain geographically, which might allow it to have a low level of gene flow with the populations of the Xiaoxinganling Mountains. (2) The marginal distribution might change the allele frequency among populations, and further affect the patterns of population structure. (3) Due to the changed climate and frequent human activity, the allele frequency are indirectly influenced by these external environment factors. However, we might be able to draw a basic preliminarily conclusion that geographic populations with similar habitats might be more likely to form the same genetic cluster. Furthermore, according to current study, we might conclude that the natural *P. koraiensis* populations in Northeast China can be divided into two groups based on the geographic distribution of this species.

The AMOVA analysis among and within populations are shown in Table S2, and the results revealed that 55.38% of the total genetic variance appeared among populations, while only 44.62% genetic variance occurred within the populations. These results indicated that most molecular variance was within populations, which was similar to the results for AMOVA analysis in the *P. koraiensis* (97.65% within populations), based on the nine EST-SSR markers [12]. Gene flow is an important factor affecting the genetic structure of plant population structure. In this study, the lowest Nm value was 0.321 between was shown between LS and HL, while the highest was observed between HL and LZH (Table S3), indicating the frequent gene flow and relatively continuous distribution pattern of the *P. koraiensis* populations.

## 4. Conclusions

In this study, we characterized and evaluated a number of EST-SSR markers derived from the transcriptome of *P. koraiensis*, and a total of twenty EST-SSR primer markers were verified with abundant polymorphisms that provide new insights into population genetics research of *P. koraiensis*. We tested the population genetic structure of natural *P. koraiensis* and found two genetic clusters. Furthermore, nearly half of these EST-SSR primer pairs were transferable across other species in the Pinus species, which would significantly reduce the cost of microsatellite marker development for *P. koraiensis* and its related species. These SSR primer pairs would provide novel genetic information for molecular breeding and could be used to accelerate the genetic improvement and breeding applications for these Pinus species.

## Figures and Tables

**Table 1:** Summary of analyses of expressed sequence tag–simple sequence repeat (EST-SSRs) in *Pinus koraiensis*.

Item	Parameters	Number
EST-SSR	Total number of sequences examined	97,376
	Total size of examined sequences (bp)	75,061,632
	Total number of identified SSRs	7235
	Number of sequences containing SSRs	6656
	Number of sequences containing more than one SSR	509
	Number of SSRs present in compound formation	354

**Table 2:** The number of repeats in each different motif length in *Pinus koraiensis*.

Number of Repeats	Mono-	Di-	Tri-	Tetra-	Penta-	Hexa-	Total	Percentage (%)
5			815	53	21	40	929	13.53
6		420	260	16	4	9	709	10.32
7		250	92	2		2	346	5.04
8		144	57	1		2	204	2.97
9		104	29				133	1.94
10	1777	68	19				1864	27.14
11	873	57	7				937	13.64
12	485	44	1				530	7.72
13	302	38	5				345	5.02
14	214	34	2				250	3.64
15	137	28					165	2.4
16	97	19					116	1.69
17	68	13					81	1.18
18	47	30					77	1.12
19	35	23					58	0.85
20	32	12					44	0.64
21	16						16	0.23
22	9						9	0.13
23	8						8	0.12
24	11						11	0.16
25	5						5	0.07
26	5						5	0.07
Others	26						26	0.38
Total	4147	1284	1287	72	25	53	6868	100
Percentage (%)	60.38	18.7	18.74	1.05	0.36	0.77		

**Table 3:** Frequencies of different repeat motifs in SSRs in *Pinus koraiensis*.

Repeats	5	6	7	8	9	10	11	12	13	14	15	16+	Total	Percentage	Rank
C/G						23	10	5	4	1	2	12	57	0.83	21
A/T						1754	863	480	298	213	135	347	4090	59.55	1
CG/CG		1	1										2	0.03	40
GC/GC		2	1										3	0.04	38
AC/GT		20	15	10	5		1	1	3	1	1	1	58	0.84	18
CA/TG		35	15	8	5	4	1	2	2				72	1.05	13
TA/TA		108	81	44	33	27	25	9	8	11	8	40	394	5.74	3
AT/AT		134	90	56	54	33	27	26	24	18	19	54	535	7.79	2
GA/TC		58	21	6	4	2	1	3				1	96	1.40	7
AG/CT		62	26	20	3	2	2	3	1	4		1	124	1.81	5
CTA/TAG	3												3	0.04	38
GTA/TAC	4		1			1							6	0.09	36
CCG/CGG	12	2	2										16	0.23	30
ACT/AGT		2											2	0.03	40
CGC/GCG	11	4	1	2									18	0.26	29
ACG/CGT	2	1		2									5	0.07	37
CGA/TCG	3	2	1			1			1				8	0.12	35
GAC/GTC	7	2											9	0.13	33
GCC/GGC	8		1										9	0.13	33
AGG/CCT	35	13	4	3	1	1	1						58	0.84	18
GGA/TCC	26	16	3										45	0.66	24
CAG/CTG	58	18	7	2	1	1		1					88	1.28	8
GCA/TGC	43	20	8	5	4	1							81	1.18	10
AGC/GCT	44	8	4	5	1		1						63	0.92	16
CTC/GAG	41	8	6	3									58	0.84	18
ATG/CAT	30	9	1	2		1	1						44	0.64	25
ACC/GGT	6	5	1	1									13	0.19	32
ATA/TAT	29	18	6	2	2								57	0.83	21
ACA/TGT	17	3	2	3	1		1			2			29	0.42	28
AAC/GTT	22	3	2	3	1				1				32	0.47	27
CAC/GTG	9	4	1	1									15	0.22	31
TAA/TTA	45	19	7	5	2	1	1						80	1.16	11
CCA/TGG	25	5	3	2	1	2	1						39	0.57	26
CAA/TTG	33	13	7	2		3			1				59	0.86	17
TCA/TGA	38	15	2	3	4	2							64	0.93	15
ATC/GAT	37	8	1										46	0.67	23
AAT/ATT	65	12	14	6	2	2	1		1				103	1.50	6
AAG/CTT	44	14	3	3	1	3	1						69	1.00	14
AGA/TCT	53	19	2		5				1				80	1.16	11
GAA/TTC	60	17	3	4	3	1							88	1.28	8
Others	114	29	4	3									150	2.18	4
Total	924	709	347	206	133	1865	938	530	345	250	165	456	6868	100	

**Table 4:** Characteristics of 20 EST-SSR loci in 80 unique genotypes in *Pinus koraiensis*.

Locus	Primer Sequence (5′–3′)	Motif	Tm (°C)	Size (bp)	Na	Ne	I	Ho	He	PIC
NFPK-218	F:AGTGGAACGAATTTGAACCG R:GGGCTTTGAAACAGGTGAAA	(TC)_6_	60	188	4	1.073	0.108	0.030	0.059	0.380
NFPK-40	F:TCGCTCTCTTCTTGACCACA R:CCGCTACTTCATCAGGGTTC	(TGA)_6_	60	196	4	1.070	0.119	0.040	0.061	0.063
NFPK-32	F:AAATGGACGAAGTTGGATGG R:CTCAGTGTCTTCAGGCAGGA	(GCT)_6_	59	197	6	2.052	0.759	0.603	0.510	0.582
NFPK-53	F:TGGAGATGCAGCAGATTAGG R:CTGCACACAGGATGTCACAA	(ATG)_6_	59	197	2	1.122	0.086	0.020	0.062	0.375
NFPK-65	F:ATGGGTATGGTGTTGGAAGG R:CTGGAGGAGCAAAATCGTGT	(TGC)_6_	59	199	11	1.451	0.491	0.325	0.281	0.588
NFPK-71	F:TTGGTGAGGATTGGTTCGAT R:CAAACTTCCGATTCGAGTGA	(AAG)_6_	60	200	5	1.957	0.725	0.622	0.484	0.405
NFPK-117	F:GCCCAATGGATGTGTCTCTT R:TCGGCCTGCAATTAGTCTCT	(TC)_12_	60	203	8	2.609	1.047	0.986	0.610	0.614
NFPK-72	F:ATCACCGCTGCCTTTCAGTA R:TCACTTCCCCAATCAATTCC	(ATA)_8_	60	204	3	1.045	0.070	0.014	0.038	0.387
NFPK-67	F:TGACCACTTCAGGCTTCTGAT R:ATGGCATCTGCTCTTTTTGC	(TGC)_6_	59	208	3	2.037	0.784	0.536	0.475	0.543
NFPK-34	F:AACCCACAGAAAGCTGAGGA R:CACCCCTGAACAGAGAGGAG	(TAA)_6_	60	221	2	0.894	0.032	0.018	0.017	0.032
NFPK-43	F:ATGCAGGGTTTGCAATACAG R:AATACGAGCACCGCGTTATC	(GAG)_6_	60	227	4	1.078	0.105	0.038	0.054	0.069
NFPK-38	F:TGATGGTGTGGTGAGGGTTA R:AGCGTGGGAGGAGTGTGTAG	(AAG)_6_	60	229	5	1.273	0.269	0.109	0.168	0.266
NFPK-150	F:AAATAACGGGGCTGTGTGTC R:ACGGATGTTGTAATCCCCAA	(GA)_6_	60	241	2	1.034	0.059	0.032	0.030	0.029
NFPK-145	F:ATGCGGAGGGATCAATTCTA R:CCAAGGCGCATCAATATTTC	(TA)_6_	60	241	14	2.985	0.989	0.296	0.513	0.794
NFPK-175	F:AAGGTCACGGCGTTCATTAC R:CCTGTGACCTCAACTGGGAT	(GA)_6_	60	259	5	1.075	0.133	0.069	0.065	0.074
NFPK-181	F:CTAAAGCGCTCAACCCAGAC R:GGACCACAGCGTGTTAGGAT	(AT)_6_	60	261	6	1.492	0.438	0.160	0.248	0.456
NEPK-179	F: CCAAGCCAGGTAAGGCACTA R: TGGACAAGGGAGATGAGACA	(CA)_10_	60	246	9	3.393	1.506	0.111	0.705	0.659
NEPK-168	F: CGGCTGTTCTGTTCCACATA R: GCCTTTGCAGTAGGATCGAG	(TA)_11_	60	261	23	3.884	2.063	0.193	0.743	0.723
NFPK-213	F:ATGTGTCACCACCCCTCATT R:ATGAGTGCGGCCTAAAGAGA	(TC)_6_	60	274	10	2.625	1.046	0.802	0.571	0.636
NFPK-184	F:AAGTCTCCACTGCATCAACCTT R:TGTCTCCCAACTTCCTGCTT	(TC)_8_	60	275	3	2.092	0.757	0.967	0.518	0.400
Mean					6.45	1.812	0.579	0.299	0.311	0.404
Total					129					

Na: number of alleles; Ne: number of effective alleles; Ho: observed heterozygosity; He: expected heterozygosity; I: information index; and PIC: polymorphic information content.

**Table 5:** Transferability in related species of Pinus using 20 EST-SSR primers developed from the cold-stressed transcriptome of *Pinus koraiensis*.

Locus	*Pinus sibirica*	*Pinus pumila*	*Pinus wallichiana*	*Pinus parviflora*	*Pinus bungeana*	*Pinus tabuliformis*	*Pinus sylvestris*	Total
NFPK-218	+	+	+	+	-	-	-	4
NFPK-40	+	+	+	+	+	+	+	7
NFPK-32	+	+	+	+	+	+	+	7
NFPK-53	+	+	+	+	+	+	+	7
NFPK-65	+	+	+	+	+	-	-	5
NFPK-71	+	+	+	+	+	-	-	5
NFPK-117	+	+	+	+	+	-	+	6
NFPK-72	+	+	+	+	+	+	+	7
NFPK-67	+	+	+	+	+	-	-	5
NFPK-34	-	-	-	-	-	-	-	0
NFPK-43	+	+	+	+	+	+	+	7
NFPK-38	+	+	+	+	+	+	+	7
NFPK-150	+	+	+	+	+	+	+	7
NFPK-145	+	+	+	+	-	+	-	5
NFPK-179	+	+	+	+	+	+	+	7
NFPK-175	+	+	+	+	-	+	+	6
NFPK-168	+	+	-	+	+	-	-	4
NFPK-181	-	+	-	-	-	-	-	1
NFPK-213	+	+	+	+	+	-	-	5
NFPK-184	+	+	+	+	+	+	+	7
Total	18	19	17	18	15	11	11	109

The “+” and “-” indicate successful and failed PCR amplification, respectively.

## Supplementary Materials

The following are available online at https://www.mdpi.com/2073-4425/11/5/500/s1. Figure S1: The results of Polymerase Chain Reaction (PCR) amplification. Table S1: Summary of eight *P. koraiensis* population information. Table S2: Analysis of molecular variance (AMOVA) results for eight populations of *P. koraiensis.* Table S3: Gene flow values between the eight populations.
