# Supplementary figures and images for: Development and Transferability of EST-SSR Markers for Pinus koraiensis from Cold-Stressed Transcriptome through Illumina Sequencing
Source: Genes (Basel). 2020 May 2;11(5):500. doi: 10.3390/genes11050500 (PMC7291311; doi:10.3390/genes11050500)

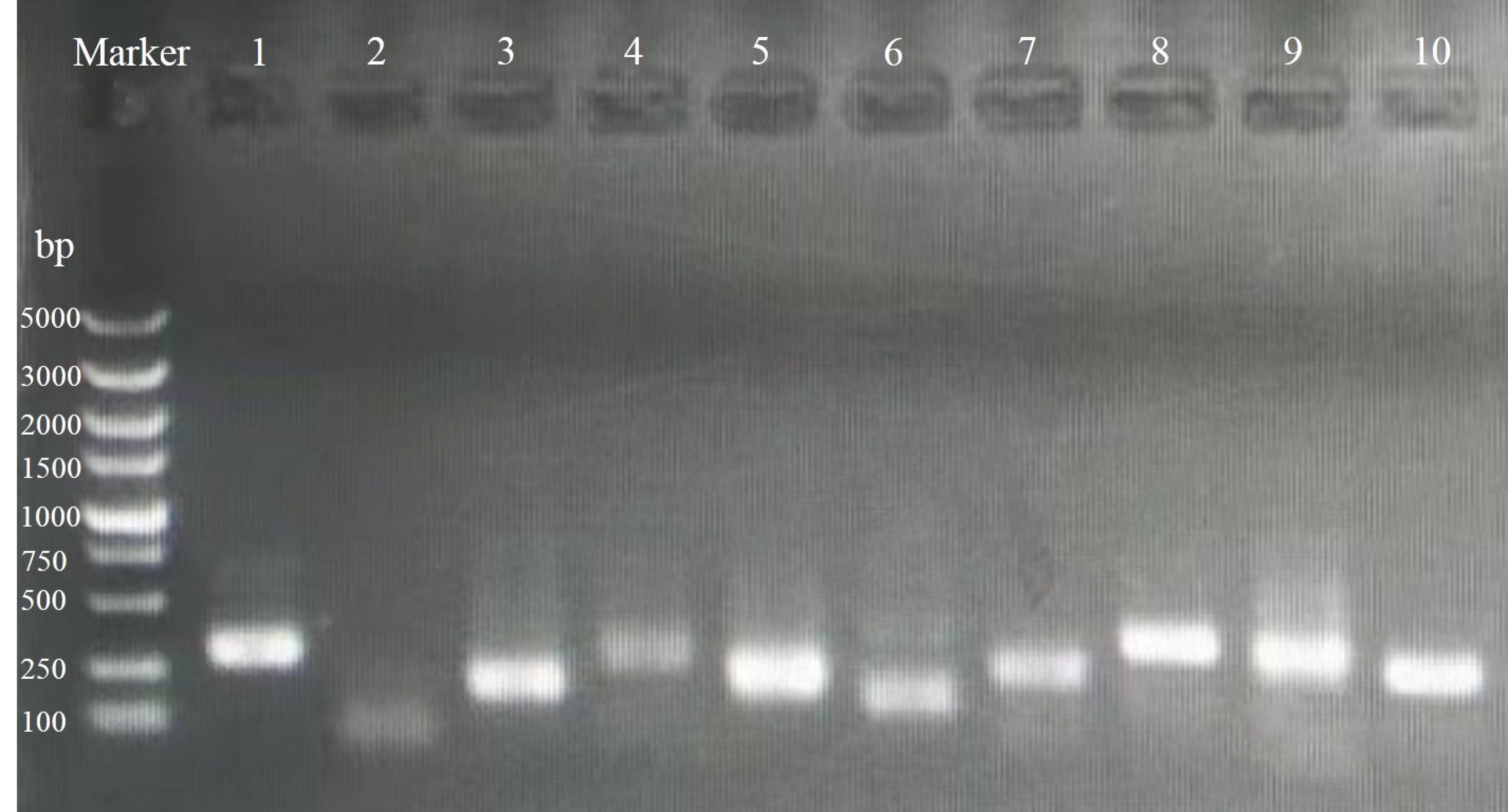

Supplement: Supplementary file 1 [file genes-11-00500-s001.zip › Supplementary files/Supplementary Figure1.jpg]
